# Central pontine myelinolysis during treatment of hyperglycemic hyperosmolar syndrome: a case report

**DOI:** 10.1186/s40842-020-00111-6

**Published:** 2020-11-16

**Authors:** Koshi Kusumoto, Nobuyuki Koriyama, Nami Kojima, Maki Ikeda, Yoshihiko Nishio

**Affiliations:** 1 Department of Diabetes and Endocrine Medicine, National Hospital Organization Kagoshima Medical Center, 8-1 Shiroyama-cho, Kagoshima, 892-0853 Japan; 2 Department of Diabetes and Endocrine Medicine, Kagoshima University Graduate School of Medicine and Dental Sciences, Kagoshima University, 8-35-1 Sakuragaoka, Kagoshima, 890-8520 Japan

**Keywords:** Central pontine myelinolysis, Osmotic demyelination syndrome, Hyperglycemic hyperosmolarity syndrome, Hypernatremia, Diffusion-weighted imaging

## Abstract

**Background:**

Central pontine myelinolysis (CPM) is a non-inflammatory demyelinating lesion of the pons. CPM and extrapontine demyelination (EPM) are together termed osmotic demyelination syndrome (ODS), a known and serious complication of acute correction of hyponatremia. Conversely, hyperglycemic hyperosmolarity syndrome (HHS) develops in patients with type 2 diabetes who still have some insulin secretory ability due to infection, non-compliance with treatment, drugs, and coexisting diseases, and is often accompanied by ketosis. HHS represents a life-threatening endocrine emergency (mortality rate, 10–50%) associated with marked hyperglycemia and severe dehydration. HHS may develop ODS, and some cases have been associated with hypernatremia.

**Case presentation:**

The patient was an 87-year-old woman with hyperglycemia, dehydration, malnutrition, and potential thrombus formation during long-term bed rest. HHS was suspected to have developed due to progression of hyperglycemia and dehydration caused by pneumonia. Furthermore, ketoacidosis developed from ketosis and prerenal renal failure associated with circulating hypovolemia shock, which was also associated with disseminated intravascular coagulation. Treatment was started with continuous intravenous injection of fast-acting insulin and low-sodium replacement fluid. In addition, ceftriaxone sodium hydrate, heparin sodium, thrombomodulin α, human serum albumin, and dopamine hydrochloride were administered. Blood glucose, serum sodium, serum osmolality, and general condition (including vital, infection/inflammatory findings, and disseminated intravascular coagulation) improved promptly, but improvements in disturbance of consciousness were poor. Diffusion-weighted imaging of the brain 72 h after starting treatment showed no obvious abnormalities, but high-intensity signals in the midline of the pons became apparent 30 days later, leading to definitive diagnosis of CPM.

**Conclusions:**

Fluctuation of osmotic pressure by treatment from hyperosmolarity due to hyperglycemia and hypernatremia in the presence of risk factors such as malnutrition, severe illness, and metabolic disorders may be a cause of CPM onset. When treating HHS with risk factors, the possibility of progression to ODS needs to be kept in mind.

## Background

Central pontine myelinolysis (CPM) with extrapontine demyelination (EPM) is called osmotic demyelination syndrome (ODS), and is now recognized as a serious complication following acute correction of hyponatremia [1]. It is believed that When osmotic pressure is rapidly increased by correction of low sodium, the blood–brain barrier (BBB) is thought to be destroyed and cytotoxic factors in the blood cause demyelination and subsequent ODS [2, 3]. However, alongside hyponatremia, many other factors are considered to be involved in the onset of ODS [4–7]. CPM is a non-inflammatory demyelinating lesion of the pons, as first reported by Adams et al. in 3 cases of chronic alcoholism and 1 case of malnutrition [4], and EPM was reported later [8].

On the other hand, hyperglycemic hyperosmolarity syndrome (HHS) develops in type 2 diabetic patients who still have some degree of insulin secretory ability due to infections, non-compliance with treatment, drugs, or coexisting diseases (endocrine diseases, cancer, etc.), and is often accompanied by ketosis. In addition, HHS is a life-threatening endocrine emergency (mortality rate, 10–50%) associated with marked hyperglycemia and severe dehydration [9]. HHS may develop to ODS [10–22], and some cases have been reported in association with hypernatremia [10–12, 15, 16].

Here, we report a rare case of ODS developing during treatment of HHS with marked hypernatremia.

## Case presentation

The patient was an 87-year-old woman with a history of venous stasis dermatitis in both lower legs. She had no history of either diagnosis of or treatment for diabetes, but hemoglobin (Hb)A1c had been recorded as 6.8% about 1 year before this presentation. She had been admitted to a psychiatric hospital for about 1 year, due to exacerbations of both depression and Alzheimer-type dementia that had developed 10 years earlier and 12 years earlier, respectively. About 2 months before presentation, her dietary intake decreased and infusion of glucose, electrolytes and water was started. She had been in a bedridden state with no speech and almost no appetite from about 1 month before presentation. At that point, hyperglycemia and hypernatremia were inferred to have already been present for a long time. Two days before presentation, sudden high fever (38 °C) and involuntary movements of the trunk and upper limbs appeared. One day later, she entered a coma. A blood glucose level (BG) of 1000 mg/dL and a serum sodium (Na) level of 179 mmol/L (glucose-corrected Na level: 194 mmol/L) were confirmed, and the patient was referred to our department for emergency hospitalization.

Glasgow coma scale score was 3 (eye opening, 1; best verbal response, 1; best motor response, 1), the pupils were 3 mm on both sides, and light reflex was rather dull, accompanied by involuntary movements of the whole body. Body temperature was 37.6 °C, blood pressure was 57/40 mmHg, heart rate was 114 beats/min, and peripheral oxygen saturation was maintained at 95% under mask administration of oxygen at 10 L/min. The tongue was very dry, and turgor of the skin was low. No abnormalities were observed in other physical findings except for the presence of moist rales at the end of inspiration in bilateral lower lung fields. Drugs being administered were limaprost alfadex at 5 mg/day, furosemide at 10 mg/day, and paroxetine at 5 mg/day.

Results of blood and biochemical examinations and blood gas analysis are shown in Table 1. Negative results were obtained for anti-glutamic acid decarboxylase antibodies (< 5.0 U/mL) (Table 1). Computed tomography of the chest showed infiltrative shadows in both lower lung fields (image not shown). This patient with hyperglycemia, dehydration, malnutrition, and potential thrombus formation during long-term bed rest was suspected to have developed into HHS and ketosis due to progression of hyperglycemia and dehydration caused by pneumonia. Furthermore, ketoacidosis had developed from ketosis and prerenal renal failure associated with circulating hypovolemia shock, which was also associated with disseminated intravascular coagulation (DIC).

**Table 1:**
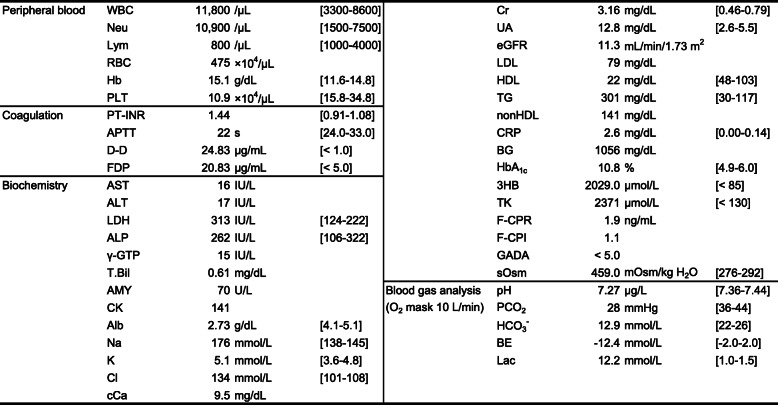
Laboratory findings on admission

For abnormal values only, reference ranges are shown in brackets

*WBC* white blood cells, *Neu* neutrophils, *Lym* lymphocytes, *RBC* red blood cells, *Hb* hemoglobin, *PLT* platelets, *AST* aspartate aminotransferase, *ALT* alanine aminotransferase, *LDH* lactate dehydrogenase, *ALP* alkaline phosphatase, *γ-GTP* γ-glutamyltransferase, *T.Bil* total bilirubin, *AMY* amylase, *CK* creatine kinase, *TP* total protein, *Alb* albumin, *Na* sodium, *K* potassium, *Cl* chlorine, *Ca* calcium, *cCa* corrected Ca, *IP* inorganic phosphorus, *Mg* magnesium, *BUN* blood urea nitrogen, *Cr* creatinine, *UA* uric acid, *eGFR* estimated glomerular filtration rate, *LDL* low-density lipoprotein cholesterol, *HDL* high-density lipoprotein cholesterol, *TG* triglycerides, *CRP* C-reactive protein, *BG* blood glucose, *HbA1c* glycated hemoglobin, *AA* acetic acid, *3HB* 3-hydroxybutyrate, *TK* total ketone bodies, *F-CPR* fasting C-peptide, *CPI* CPR index, *GADA* anti-glutamic acid decarboxylase antibody, *sOsm* serum osmolality, *BE* base excess, *Lac* lactate

**Fig. 1:**
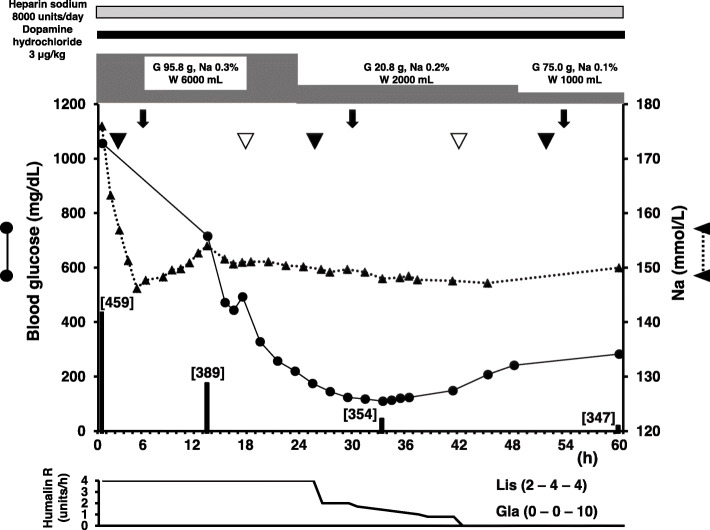
Clinical course. The clinical course up to 60 h after admission. Closed circles and solid lines show changes in blood glucose levels, and closed triangles and dotted lines show changes in serum sodium. Down arrow indicates infusion of 6400 units of thrombomodulin α. Closed and open arrowheads indicate infusions of 1 g of ceftriaxone sodium hydrate and 12.5 g of human serum albumin, respectively. Closed bar and numbers in brackets indicate serum osmolality. The line graph at the bottom shows insulin usage. Numbers in parentheses indicate, from left to right, the number of insulin units used just before breakfast, just before lunch, and just before dinner. G, glucose content; Na, sodium concentration; W, amount of water; Lis, insulin lispro; Gla, biosimilar insulin glargine

**Fig. 2:**
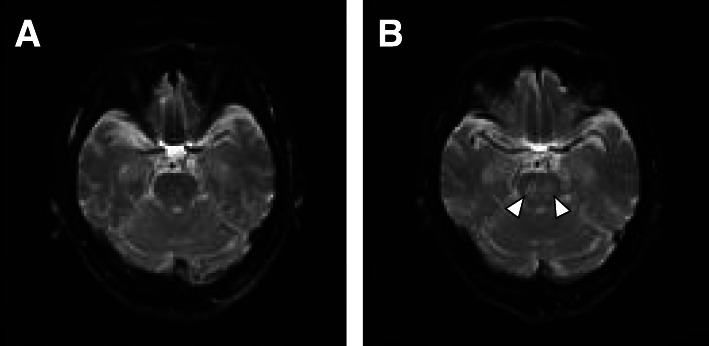
Diffusion-weighted imaging of the brain. **a** Image 72 h after admission. **b** Image 30 days after admission. Arrowheads indicate high-intensity regions in the pons

Treatment was started with **i**ntravenous infusion of fast-acting insulin (Humalin R; Eli Lilly, Kobe, Japan) (starting at 4 units/h and gradually decreasing) and low-sodium replacement fluid [23]. In the first 24 h, 6000 mL of replacement fluid (95.8 g of glucose, 0.3% Na) was added, and 2000 mL of replacement fluid (20.8 g of glucose, 0.2% Na) was administered within the period of 24–48 h. At 48–72 h, 1000 mL of replacement solution (75 g of glucose, 0.1% Na) was administered, and combined use of tube feeding was started (Fig. 1). Correction of K was performed appropriately. Although BG was ≥ 1000 mg/dL at 8 h after starting treatment, Na improved to 149.5 mmol/L (glucose-corrected Na level: 164.4 mmol/L). After 24 h, although BG, Na and sOsm had decreased to 716 mg/dL, 154.0 mmol/L and 402.3 mOsm/kg H_2_O, respectively, glucose-corrected Na level remained almost unchanged (164.2 mmol/L). At 48 h later, BG had improved to 110 mg/dL, Na to 154 mmol/L, and sOsm to 370.0 mOsm/kg H_2_O. However, glucose-corrected Na level (166.0 mmol/L) was not showing improvement. At 72 h later, BG had improved to 283 mg/dL, Na to 150 mmol/L (glucose-corrected Na level: 152.5 mmol/L), and sOsm to 345.5 mOsm/kg H_2_O (Table 2). In addition, ceftriaxone sodium hydrate at 1 g/day, heparin sodium at 8000 units/day, thrombomodulin α at 6400 units/day, total human serum albumin at 62.5 g, and dopamine hydrochloride at 3 μg/kg were administered. General condition, including vital signs, infection/inflammatory findings, and DIC improved promptly (Fig. 1). After 72 h, the patient opened her eyes. However, because the state of no response to the stimulus and involuntary movements continued for 7 days, ODS was suspected and magnetic resonance imaging (MRI) of the brain was performed. No clear abnormalities were evident on diffusion-weighted imaging (DWI) (Fig. 2a), and no definitive diagnosis was reached. The patient was subsequently able to move in response to instructions, but we could not exclude the possibility of pseudobulbar paralysis associated with ODS, as she could barely speak and showed no improvement of dysphagia. A high-intensity signal in the pons was identified on DWI of the brain at 30 days after starting treatment (Fig. 2b), leading to definitive diagnosis of CPM. After the life-threatening state was averted and general condition improved, she was transferred to a long-term care facility. As of about 1 year after onset, we obtained information that the patient had recovered to the point that she could speak spontaneously and responded to conversation in a manner reflective of a good condition.

**Table 2:** Changes in glucose, serum sodium, glucose-corrected serum sodium and serum osmotic pressure due to acute treatment

Time from start of treatment (h)	Glucose (mg/dL)	Sodium (mmol/L)	Glucose-corrected sodium (mmol/L)	Osmotic pressure (mOsm/kg H_2_0)
0	1000	179.0	194.0	488.2
8	1000	149.5	164.4	429.0
24	716	154.0	164.2	402.3
48	110	154.0	166.0	370.0
72	283	150.0	152.5	345.5

## Discussion and Conclusions

Although ODS has been reported to rarely develop in HHS [10–22], fewer reports have described development of ODS in HHS with hypernatremia [10–12, 15, 16], and causes of ODS development have yet to be clarified in terms of marked hyperosmolarity or changes in osmotic pressure associated with treatment. Within 7 days of onset, findings of ODS are not detectable on MRI [24, 25]. On the other hand, Ruzek et al. reported DWI as extremely useful for early diagnosis of CPM, based on the possibility of diagnosing CPM by MRI 24 h after onset [26]. MRI findings, including DWI, are also known to be observed 24 h after onset [25, 27]. In this case, no clear abnormality was observed on DWI at 72 h after symptom onset. Relatively rapid improvement (fluctuation) of osmotic pressure by treatment for hyperosmolarity due to hyperglycemia and hypernatremia was thought to be the cause of CPM onset in this case. A similar case in which CPM and EPM developed due to rapid improvement of hypernatremia was reported by Go et al. [28].

The issue that we would have changed in our treatment of this case was the performance of dehydration correction using a hypotonic solution. We speculated that rapid changes in osmotic pressure might have been avoidable using physiological saline or at least half-saline. On the other hand, slowly reducing blood glucose may be a meaningful strategy from the perspective of preventing the onset of ODS. Regarding the pathogenesis of ODS, rapid changes in osmotic pressure presumably induce apoptosis of astrocytes [2] and disrupt the blood–brain barrier. As a result, cytotoxic factors in blood become able to enter the brain, injuring oligodendrocytes and leading to demyelination [3]. Furthermore, microglia reportedly activate early in the onset of ODS and accumulate in the demyelinating region, and may express inflammatory cytokines and participate in the progression of demyelination, leading to “myelin melting” [29]. With these mechanisms, dexamethasone reportedly acts to prevent breakdown of the blood–brain barrier, while minocycline may prevent the onset and development of ODS by suppressing the expression of inflammatory cytokines from microglia and migration and accumulation of microglia to demyelinated parts; that is, by suppressing microglial activation [30]. Dexamethasone and minocycline may thus have potential as clinical therapeutic agents for ODS in the future. However, the use of dexamethasone in patients with severe hyperglycemia requires careful consideration and may not represent a suitable first-line option for the treatment of DOC. Further, alcohol poisoning, liver diseases including liver transplantation, malnutrition, malignant tumors, severe diseases or sepsis during pregnancy or postpartum, adrenal insufficiency, and metabolic disorders have been mentioned as risk factors for the development of ODS [7]. Our patient also showed malnutrition, severe illness, and metabolic disorders as risk factors.

A key limitation of this report was that the condition of the patient before admission to our hospital could not be accurately gauged due to a lack of data from the referring facility. In addition, the interval to follow-up MRI was about 1 month, providing a weak basis for estimating the time of ODS onset.

In conclusion, we have reported a rare case involving an 87-year-old woman with CPM during treatment of HHS with marked hypernatremia. Fluctuations in osmotic pressure with treatment for hyperosmolarity due to hyperglycemia and hypernatremia in the presence of risk factors such as malnutrition, severe illness, and metabolic disorders were considered as the causes of CPM onset. When treating HHS with risk factors, the possibility of progression to ODS should always be kept in mind.

## Data Availability

Not applicable.

## Abbreviations

CPM: Central pontine myelinolysis
EPM: Extrapontine demyelination
ODS: Osmotic demyelination syndrome
HHS: Hyperglycemic hyperosmolarity syndrome
BG: Blood glucose level
Na: Sodium
K: Potassium
sOsm: Serum osmolality
DIC: Disseminated intravascular coagulation
MRI: Magnetic resonance imaging
DWI: Diffusion-weighted imaging
